# College Notes

**Published:** 1894-02

**Authors:** 


### College Notes.

### A FOUR YEARS' COURSE AT JEFFERSON MEDICAL COLLEGE.

At a meeting of the faculty of Jefferson Medical College, held on January 8, 1894, it was unanimously resolved to institute a compulsory four years' course with the session of 1895–96. This step was taken in order that the large clinical service of the Jefferson College Hospital (350 cases a day) might be utilized to the fullest extent in carrying out the desire of the faculty to provide advanced medical education of a practical character.

### HIGHER MEDICAL EDUCATION AT RUSH.

In pursuance of the policy recently announced in the resolution to be presented to the American Medical College Association, the trustees and faculty of Rush Medical College have decided to require four years' attendance at college from students who begin the study of medicine this year, with a view to graduation in 1898. However, those who have already studied medicine one year or more with a preceptor, so that the four years of study already required will be completed before July, 1897, may graduate after three courses of lectures as heretofore. To encourage proper preliminary study, graduates in arts and sciences from high grade colleges, and graduates in pharmacy and dentistry from colleges requiring a proper amount of study and two full courses of lectures, will, until further notice, be allowed to graduate after an attendance on only three courses of lectures.

